# When Is a New Scale not a New Scale? The Case of the Bergen Shopping Addiction Scale and the Compulsive Online Shopping Scale

**DOI:** 10.1007/s11469-016-9711-1

**Published:** 2016-10-27

**Authors:** Mark D. Griffiths, Cecilie S. Andreassen, Ståle Pallesen, Robert M. Bilder, Torbjørn Torsheim, Elias Aboujaoude

**Affiliations:** 1 International Gaming Research Unit, Psychology Department, Nottingham Trent University, Nottingham, UK; 2 Department of Clinical Psychology, University of Bergen, Bergen, Norway; 3 Department of Psychosocial Science, Bergen, Norway; 4 Norwegian Competence Center for Sleep Disorders, Bergen, Norway; 5 Semel Institute for Neuroscience and Human Behavior, University of California, Los Angeles, Los Angeles, CA USA; 6 OCD Clinic, Department of Psychiatry and Behavioral Sciences, Stanford University School of Medicine, Stanford, CA USA

**Keywords:** Bergen shopping addiction scale, Shopping addiction, Compulsive buying, Compulsive shopping, Psychometric scales, Behavioral addiction

## Abstract

Manchiraju et al. (*International Journal of Mental Health and Addiction*, 1–15, 2016) published the Compulsive Online Shopping Scale (COSS) in the *International Journal of Mental Health and Addiction* (*IJMHA*). To develop their measure of compulsive online shopping, Manchiraju and colleagues adapted items from the seven-item Bergen Shopping Addiction Scale (BSAS) and its’ original 28-item item pool. Manchiraju et al. did not add or remove any of the original seven items, and did not substantially change the content of any of the 28 items on which the BSAS was based. They simply added the word “online” to each existing item. Given that the BSAS was specifically developed to take into account the different ways in which people now shop and to include both online and offline shopping, there does not seem to be a good rationale for developing an online version of the BSAS. It is argued that the COSS is not really an adaptation of the BSAS but an almost identical instrument based on the original 28-item pool.

Over the last few decades, research into ‘shopping addiction’ and ‘compulsive buying’ has greatly increased (Aboujaoude 2014; Maraz et al. 2016). In 2015, we developed and subsequently published a new scale to assess shopping addiction – the 7-item Bergen Shopping Addiction Scale (BSAS) (Andreassen et al. 2015). We noted in that paper that two scales had been developed in the 2000s (i.e., Christo et al. 2003; Ridgway et al. 2008), but that neither of these instruments approached problematic shopping behavior as an addiction in terms of core addiction criteria that are often used in the behavioral addiction field including salience, mood modification, tolerance, withdrawal, conflict, relapse, and problems (Griffiths 2005). We also noted that new Internet-related technologies can greatly facilitate the emergence of problematic shopping behavior because of factors such as accessibility, affordability, anonymity, convenience, and disinhibition (Aboujaoude 2011; Griffiths 2003), and that there was a need for a psychometrically robust instrument that assesses problematic shopping across all platforms (i.e., both online and offline). We concluded that the BSAS has good psychometrics, structure, content, convergent validity, and discriminative validity, and that researchers should consider using it in epidemiological studies and treatment settings concerning shopping addiction.

More recently, Manchiraju et al. (2016) published the Compulsive Online Shopping Scale (COSS) in the *International Journal of Mental Health and Addiction* (*IJMHA*). Given that we had just developed a new shopping addiction scale that covered shopping across all media, we were interested to read about the new scale. The scale was a 28-item scale and was based on the 28 items included in the first step of BSAS development (i.e., initial 28-item pool). As the authors noted:

*“First, to measure compulsive online shopping, we adopted the Bergen Shopping Addiction Scale (BSAS; Andreassen et al.* 2015 *). The BSAS developed by Andreassen et al. (* 2015 *), was adapted for this study because it meets the addiction criteria (e.g., salience, mood modification, etc.) established in the DSM-5. In total, 28 items from the BSAS were modified to reflect compulsive online shopping. For example, the original item – ‘Shopping/buying is the most important thing in my life’ was modified as ‘Online shopping/buying is the most important thing in my life’… It is important to note that we are proposing a new behavioral addiction scale, specifically compulsive online shopping … In conclusion, the scale developed in this study demonstrated strong psychometric, structure, convergent, and discriminant validity, which is consistent with Andreassen et al.’s (* 2015 *) findings”.*

Apart from the addition of the word ‘online’ to every item, all initial 28 items of the BSAS were used identically in the COSS. Therefore, we sought the opinion of several research colleagues about the “new” scale. Nearly all were very surprised that an almost identical scale had been published. Some even questioned whether such wholescale use might constitute plagiarism (particularly as none of the developers of the COSS sought permission to adapt our scale).

According to the plagiarism.org website, several forms of plagiarism have been described (2016), including: *“Copying so many words or ideas from a source that it makes up the majority of your work, whether you give credit or not”* (p.1). Given the word-for-word reproduction of the 28 item–pool, an argument could be made that the COSS plagiarizes the BSAS, even though the authors acknowledge the source of their scale items.

According to Korb’s (2012) article on adopting or adapting psychometric instruments:

*“Adapting an instrument requires more substantial changes than adopting an instrument. In this situation, the researcher follows the general design of another instrument but adds items, removes items, and/or substantially changes the content of each item. Because adapting an instrument is similar to developing a new instrument, it is important that a researcher understands the key principles of developing an instrument…When adapting an instrument, the researcher should report the same information in the Instruments section as when adopting the instrument, but should also include what changes were made to the instrument and why”* (p.1).

Manchiraju et al. did not add or remove any of the original seven items, and did not substantially change the content of any of the 28 items on which the BSAS was based. They simply added the word “online” to each existing item. Given that the BSAS was specifically developed to take into account the different ways in which people now shop and to include both online and offline shopping, there does not seem to be a good rationale for developing an online version of the BSAS. Even if there was a good rationale, the scale could have made reference to the Bergen Shopping Addiction Scale in the name of the ‘new’ instrument.

Oakland (2005) also notes the following in relation to plagiarism and psychometric test development:

*“Psychologists do not present portions of another’s work or data as their own, even if the other work or data source is cited … Plagiarism occurs commonly in test adaptation work (Oakland and Hu* 1991 *), especially when a test is adapted without the approval of its authors and publisher. Those who adapt a test by utilizing items from other tests without the approval of authors and publishers are likely to be violating ethical standards. This practice should not be condoned. Furthermore, this practice may violate laws in those countries that provide copyright protection to intellectual property. In terms of scale development, a measure that has the same original items with only one word added to each item (which only adds information on the context but does not change the meaning of the item) does not really constitute a new scale. They would find it really hard to demonstrate discriminant validity between the two measures”.*

Again, according to Oakland’s description of plagiarism specifically in relation to the development of psychometric tests (rather than plagiarism more generally), the COSS appears to have plagiarized the BSAS particularly as Oakland makes specific reference to the adding of one word to each item (“*In terms of scale development, a measure that has the same original items with only one word added to each item … does not really constitute a new scale”*).

Still, it is important to point that we have no reason to think that this use of the BSAS was carried out maliciously. Indeed, it may well be that the only wrongdoing was lack of familiarity with the conventions of psychometric scale development. It may be that the authors took one line in our original paper too literally (*“the BSAS may be freely used by researchers in their future studies in this field”*). However, the purpose of this sentence was to give fellow researchers permission to use the validated sale in their own studies and to avoid the inconvenience of having to request permission to use the BSAS and then waiting for an answer. Another important aspect here is that the BSAS (which may be freely used) consists of seven items only, not 28. The seven BSAS items were extracted from an initial item pool in accordance with our intent to create a brief shopping addiction scale. Consequently, there exists only one version of BSAS, the 7-item version. Manchiraju et al. (2016) seem to have misinterpreted this when referring to a 28-item BSAS.

A final point to raise is the failure of the original reviewers of the paper by Manchiraju et al. to spot that the COSS was not really an adaptation of the BSAS but, rather, an almost identical instrument based on the 28-item pool. This highlights the critical importance of the review process and the centrality of the reviewers’ role in upholding the ethics and conventions of scientific research. In this, there is a lesson for all reviewers, authors and, indeed, journals and editors.
